# Abstracts and Gleanings

**Published:** 1884-08-20

**Authors:** 


### ABSTRACTS AND GLEANINGS.

**Advice to the Medical Witness.**—Dr. Strong, in a paper before the Oregon State Medical Society, writes: "As an expert, the physician will frequently be called upon for his opinion, and, as his conclusions are to be deduced from proven facts, they must be carefully drawn to possess any value. To perform this duty thoroughly, he should, therefore, not wait until in the witness box. Tidy's advice may well apply at this point. He says: 'And if, in the quiet of your study, you fail to come to a satisfactory conclusion, do not attempt a wild conjecture in the hurry and excitement of the witness box. To *be* accurate is ten thousand times better than to *appear* brilliant.'

"The physian should carefully study the opinions held and expressed by others, and be able to give good definite reasons why he adopts some and rejects others, always remembering that he will be exposed to the scathing fire of cross-examination.

"He should bear in mind the difference between a fact and an opinion, so that there may be no confusion in his mind regarding their identity. For example, it is a fact that certain drugs are deadly poisons; but their action in producing certain effects is an opinion. The direction, size and character of a wound are facts. Deductions drawn as to the manner in which the wound was produced, or for what purpose, is, in most cases, a matter of opinion. An opinion, however, is always based on facts, and either a personal knowledge of the circumstances relating to these facts or knowledge gained from undisputed authority concerning them is essential. No tolerance can be given to hearsay or rumor.

"A bias statement given by a witness is invariably detected, and an attempt of the witness to arrogate to himself any of the duties of the jury injures the value of his evidence.

"The plainest English possible should be employed, and any tendency to exaggeration suppressed. Be sure before answering that the entire question is thoroughly understood, and the question alone asked should be answered without ambiguity or useless expressions. All 'ifs' and 'thats' should be omitted, if possible, and the answers should convey real meaning in such clear, unmistakable language that there can be no misunderstanding.

"If no distinct opinion on a certain subject has been formed, there should be no hesitation in saying so; and the physician should never allow himself to be drawn into, or give, an opinion formed on the spur of the moment in the witness box.

"As nearly as possible, the exact language of conversations testified to, or authorities quoted, should be given.

"When the close pressure of cross-examination occurs, the only safety of the witness is in coolness, self-possession and a thorough knowledge of the case. If he lose his temper, he is sure to be led on until he irretrievably damages himself, his testimony, or his medical reputation.

"Admitted ignorance of a question not understood is not only not condemnatory, but praiseworthy; and, within certain limits, the answer 'I do not know' is both safe and honorable.

"A witness may be obliged to answer yes or no in a given case; but, though he may not modify it, he has a right to explain his answer to make it comprehensible; and he should always avail himself of that privilege, to prevent any chance of a misunderstanding of his meaning. All facts should be given as the witness understands them, without reference as to their effect, and in opinions drawn from facts, if any honest doubts arise, they should be plainly stated.

"The witness should never allow himself to be drawn into a discussion; but, having given an opinion, and the reason for it, let it rest there. He is entitled to have the question fairly and clearly stated to him, and the utmost care is required that the conditions of a hypothetical case should be plainly discerned and properly understood by him before answering. If the hypothetical contain impossibilities or inconsistencies, he should never endeavor to give a mixed answer, but insist that a proper case be given him.

"One of the most important points of all to be remembered is, that the opposing attorney will probably attempt to impair the value of important testimony given by the medical witness, by showing lack of professional knowledge, and will propound questions which are incapable of definite answers, because of differences of opinion among high medical and legal authorities. The only manner by which such an attack can be met is to enter the court room prepared to state the existence of such differences, when they exist, and as they will, probably, relate, either in a direct or remote manner, to the subject of trial, the simple form of preparation is that recommended by Tidy, namely, get the case well up in your office before the trial."

**Chloroform as an Anti-Ferment.**—On one occasion a relative of mine wanted a bottle to put milk in to keep it sweet for her child, which was subject to colics and very offensive discharges of curdled milk from the bowels. Looking over my stock of bottles, I found a chloroform bottle containing about ten drops of chloroform. Thinking that the chloroform might benefit his colic, I filled the bottle with milk, shook it up, and used to good advantage. Some of the milk was left, and in the evening I found that it was still sweet after standing ali day in a warm place. The next day it was sweet. The next week it was sweet! The next month it was sweet!! I concluded to leave it among my medicines indefinitely to see when it would decompose. The bottle was stopped tightly to prevent evaporation of the chloroform, only opening it occasionally to examine the condition of the milk. I finally lost signt of it. After three or four years I accidentally came across it and found it still as left on last examination. I have come to the conclusion that one drachm of chloroform will preserve a pint to one quart of milk indefinitely, if securely stopped to prevent evaporation of the chloroform.—*Med. World.*

**Traumaticin as a Medium for the Application of Certain Substances in Diseases of the Skin.**—Considerable attention has been given lately to the application of remedies to the skin in adhering layers. Prof. Pick was the first to point out the use of gelatin for this purpose. Prof. Auspitz advocates the use of traumaticin (which corresponds to the liquor Gutta Perchæ, B. P.) as a vehicle for chrysarobin in preference to gelatin. The gelatin layer peels off in a few hours, and must be re-applied. Traumaticin forms a much thinner and more delicate covering than collodion or gelatin, and for this reason causes neither tension nor pain. Traumaticin is, of itself, a neutral covering and protective remedy. Ten per cent. solutions, even when applied to large surfaces on children or on adults, have never caused irritation. It also causes much more uniform pressure than gelatin, is much more easily manipulated, and does not decompose. After the greater part of the scales have been removed by a soap bath, a ten per cent. solution is applied with a stiff bristle brush to all the existing patches. When the eruption is limited to a few and small patches, the solution is applied daily; when large surfaces are involved, every second or third day. A previous soap bath, when the reproduction of scales is very great, or simply a local wash with soap, when the scales are scanty, is advisable. Many patches, after one or two applications, already appear a great deal flatter, and are covered, mostly around the borders only, with scales. After two, at most twelve, applications the infiltration and scales disappear, and in their place white spots, bounded by a red or violet-brown line, appear. In a few cases of limited extent the psoriatic process was stopped, in from three to six days, by daily application, without bathing or washing. In spite of the frequent application of the remedy to the face, and, in psoriasis universalis, to almost the whole external surface of the body, no inflammatory conditions of the healthy tissues—in the form of conjunctivitis, diffused redness, painful swelling, acne, furuncles, eczema, or as fever, or disturbances of the whole general organism—were observed. Parasitic diseases, herpes tonsurans, eczema marginatum and prurigo can be treated in the same manner. In prurigo, chrysarobin traumaticin was applied in ten per cent. solutions to adults, and in five per cent. solutions to children. Usually the itching was immediately alleviated. In a few cases, after two to six applications, the nodules had disappeared. That chrysarobin, however applied, does not prevent the recurrence of the disease need not be mentioned; and the word *cured* above is only to be understood as meaning that the existing patches had disappeared. Traumaticin is as good an excipient for pyrogallic acid and for salicylic acid as for chrysarobin.—*Prof. Auspitz, Dublin Med. Journal, Feb., p. 189.*

**Opacities of the Cornea.**—Dr. Michel reeommends sulphate of cadmium, of the strength of two and a half grains to the ounce of mucilage, as an application to opacities of the cornea. A camel's-hair brush, dipped in this wash, is applied to the centre of the spot and retained in contact with it for a few seconds. At first the application is made once a day, but after a while is repeated two or three times in the twenty-four hours. When the pain grows less, the strength of the solution may be increased to five grains or even seven grains to the ounce. When the opacity is of recent formation it readily disappears under this treatment, but when it is of old date the applications must be long-continued.—*Practitioner, Jan., p. 53.—Braithwaite's Retrospect.*

**Painful Ulcers of the Cornea.**—The treatment by warmth and eserine is very easily carried out. The patient is directed to foment the eye for fifteen or twenty minutes three or four times a day, or oftener. The fomentation should be used as hot as can be borne, and may consist of simple hot water, decoction of poppy-heads or cahmomiles. A solution of one or two grains of sulphate of eserine to an ounce of water should be dropped into the eye three or four times a day, after the fomentations have been used. A large pad of cotton-wool, thoroughly warmed before a fire or over a spirit-lamp, should be laid upon the closed eyelids and secured by a bandage, and replaced by a freshly warmed one as often as may be necessary for the patient's comfort. The patient should be allowed to go about; dark rooms and confinement to the house always make matters worse. He should be well fed, purged if necessary, and placed on some tonic, none being better than iron in some form or other. Stimulants are rarely needed, and in many cases will do harm. The treatment by warmth without eserine is most useful in many painful inflammations of the eyes. It was first, I believe, recommended by Mr. Liebreich.—*Mr. Charles Higgens, p. 144.—Braithwaite's Retrospect.*

**Strychnia in Delirium Tremens.**—Dr. Dujardin-Beaumetz (Bulletin de Therapeutique), while disagreeing with the opinion of Dr. Luton, of Rheims, that strychnia is the appropriate medical agent for combating alcoholism in general, is quite in accord with him as to its great value in the treatment of delirium tremens. In this it is one of the most certain and efficacious of remedies, which he has successfully availed himself of in many cases at the St. Antoine Hospital. He administers it in hypodermic injections, beginning with a dose of five milligrams, which he repeats in five hours. Sometimes, if the symptoms persist, he gives a third injection within the twenty-four hours.—*Medical Press.*

**To Prevent Necessity for Inducing Abortion.**—The Obstetric Gazette, quoting from Deutsche Med. Zeit., says that Depaul recommends in all such cases of contracted pelvis which indicate the necessity for inducing premature labor, that an attempt be made to lessen the size of the child, by administering iodide of potassium to the woman.

He claims that this treatment was successful in a case of his, in which the conjugate of the true pelvis was from 7½ to 7¾ ctm. The previous three labors, in which she had no treatment, were very tedious and exhausting, while in this one labor came on at term, spontaneous and easy, the child living.—*Med & Surg. Jour.*

**Rapid Absorption of Pleuritic Effusion by use of Salt and Cutting-off Liquids.**—Tom Robinson, M.D., (in British Medical Journal, Dec. 22, 1883, p. 1234,) says: "John C. came under my observation on August 1st, 1882. He was then suffering from a well-marked left pleurisy, *i. e.*, pain, catching breathing and a cry at the end of inspiration. He sat up in bed and held his hand against his side, complaining bitterly of the pain. A bandage was put firmly round the thorax, with much and rapid relief, and he was ordered absolute rest, a liquid diet and some opium. On August 2d he was evidently much relieved, and his respiration was more uniform, deeper and attended with less pain.

"On August 3d I was hastily sent for, and, on my arrival, I found my patient in great and urgent distress. His lips were livid, his nostrils were smoky, his eyes were prominent and his nails blue. He had cold clamminess of hands and brow, and he was breathing fifty-one times in a minute. The pulse was barely appreciable at his wrist. The heart was pushed over to the right. I told him of his great danger, and urged him to permit me to draw off the fluid. This he flatly refused to allow me to do, saying that a friend of his was tapped, and died one hour afterward. This was at 11 a. m. I put him under the following conditions: He was to take every hour one teaspoonful of common salt, dissolved in a wine-glassful of tepid water. I produced sweating by a hot wet flannel and a piece of water-proof sheeting, and I gave him two ounces of the common black draught at once, and stopped all fluids. At 1 p. m. he said: "I can get my wind now," and he had most markedly improved in every way. He looked less livid. His respiration had dropped down to forty eight. At 9 p. m. he looked cheerful. The lividity had almost disappeared. He could lie on either side and get well down in the bed.

"On August 4th. on entering the room, I could not have detected any embarrassment of breathing, and all his subjective symptoms had disappeared. There was comparative dullness in the left base, but the air could be heard entering the lung. I now gave him one drachm of common salt twice a day and two ounces of the brandy-mixture of the Pharmacopœia every four hours, together with some oysters and anchovy and Digby chick or a piece of salted bacon. He did not complain very much of the thirst; said his "mouth was dry," and his tongue looked red and glazed. From this time there is practically nothing to say. My patient never had a return of his symptoms, and he was up and out of doors in a week.

"It is barely necessary to comment upon the rationale of the treatment adopted in this case, and my only object in bringing it forward is to see whether like results will follow in the experience of other similar cases, or in any effusion which takes place into a serous cavity. The salt, I believe, increased the density of the blood; the blood became thirsty, if I may so express it, and drank up the pleuritic effusion in a manner to me singularly rapid and striking. Doubtless the sweating and purging aided me very much; but purging and sweating will not remove serous effusion with precision."—*Brit. Med. Jour., Dec. 22, 1883, p. 1234.*

**Enemas in Severe Forms of Constipation.**—"Let me recommend O'Beirne's classical treatise on defæcation to those who are unacquainted with it. I do not think I have ever reaped more practical profit from any of my medical reading than from a study of its pages. The gist of O'Beirne's book is the recommendation of the long enema-tube which, for fifty years, has been known by his name. Never entrust the use of O'Beirne's tube to a nurse. The efficient passage of this instrument into and through the sigmoid flexure of the colon is a delicate and difficult operation, which the medical attendant ought always himself to perform for his patient. Much unnecessary detail has been taught about the composition of enemata. When we use an enema for the purpose of clearing the bowel of fæces and flatus, the quantity of the injection is its chief quality. I am accustomed to tell my pupils that, when they give an enema, they should always ask themselves whether it is to be retained or returned. If it be designed that the injection shall be retained, as in the case of a nutrient and sedative enema, its quantity can scarcely be too small; if, on the other hand, it be intended to move the bowels to the expulsion of their contents, the quantity of an enema can scarcely be too large. The quantity of an aperient injection is precisely as much of it as can be passed into the bowel. For such an enema to be as large as possible, is only to be large enough."—*British Medical Journal, Nov. 17, 1883, p. 905.*

**Nasal Hemorrhage.—Plugging the Nose.**—For the arrest of nasal hemorrhage I know of no device so good as one that may be readily extemporised with a strong piece of cord and some small pieces of sponge. The cord is tied securely to a piece of sponge, cut rounded, and just large enough to be forced backward through the nostril. Then a number of similar pieces of sponge, with a hole through the centre of each, are threaded successively on the cord. The sponge on the end of the cord is then pushed, with a probe or dressing forceps, through the nostril, quite back to the faucial orifice, and the rest of the threaded pieces of sponge are slid back, one at a time, until the nares are tightly filled. When the patient becomes secure against a repetition of hemorrhage, the plugging is readily removed, one piece of sponge being withdrawn at a time with the dressing forceps.—*Dr. R. J. Levis, Pennsylvania, Practitioner, Nov., p. 380.—Braithwaite's Retrospect.*

**To Abort a Stye.**—Dr. Louis Fitzpatrick, who has recently returned from Egypt, where all kinds of eye affections are extremely common, writes to the Lancet that he has never seen a single instance in which the stye continued to develop after the following treatment had been resorted to. The lids should be held apart by the thumb and index finger of the left hand or a lid-retractor, if such be at hand, while the tincture of iodine is painted over the inflamed papilla with a fine camel's hair pencil. The lids should not be allowed to come in contact until the part touched is dry. A few such applications in the twenty-four hours are sufficient.—*Amer. Drug.*

**Case of Uterine Disease.**—Dr. Bentley writes (Ther. Gaz.) that he gave the annexed treatment in a case in which the most prominent features were habitually constipated bowels, feeble and capricious appetite, very prominent indigestion, frequent fits of persistent vomiting, lasting, at times, for several days, and at catamenial periods a long series of distressing hysterical convulsions, with fixed gaze, locked jaws, difficult breathing and deglutition, and insensible voiding of urine and fæces. A most offensive leucorrhœa was persistent—always present.

On examination, I found the uterus much enlarged, cervix elongated and pointing, some prolapsus with anteversion, vagina almost occluded by a "velvety growth," the product of inflammation; uterus and vagina much inflamed and extremely sensitive.

I proceeded at once to unload the bowels and correct their languor with the following:

℞ Fl. ext. nux . . . . . . . . . . ʒ ij,

Fl. ext. wahoo . . . . . . . . . . ʒ vj,

Fl. ext. cascara sagrada . . . . . . . . ℥ j.

Mix. Sig. Take twenty-five to thirty drops three times a day.

The bowels were soon corrected. I then gave F. F. Burdock seeds, at first in half-drachm doses, which were gradually increased to drachms, three times a day before meals, and an additional dose at bed time.

Locally, I used tampons of cotton wool. First, the cotton was well washed with soap and water, then dried and well carded. I then thoroughly saturated the cotton in the following mixture:

Powdered alum . . . . . . . . . . grs. lx,
Powdered borax . . . . . . . . . . grs. v,
Hot water . . . . . . . . . . f ℥ ss,
Glycerine . . . . . . . . . . f ℥ jss.

With this cotton the vagina was thoroughly packed, taking care to put the uterus in proper position. The tampon was carefully removed, the vagina well washed with soap suds and refilled, every two or three days.

Improvement was almost immediate and complete recovery rapid. She is now, June 17th, 1884, after two months' treatment, entirely well.

I ascribe much, but not all, to the burdock. The cascara mixture was certainly an important factor, and last, though not least, the local measures—so I think.

**The Poison of the Skunk.**—Dr. Howard Jones, of Circleville, Ohio, after careful inquiry throughout some of the Western States, during which he collected the histories of fifty-two cases of bites from the common skunk, arrives at the following conclusions: That the bite of the common skunk is, under certain conditions, dangerous, and even extremely fatal, there can be no doubt; and there are many reasons for believing that the disease is identical with rabies in the dog or hydrophobia in man.—*South. Clinic.*

**Nurses' Sore Mouth.**—A writer in American Medical Journal says: "In all cases of nursing sore mouth, there may be found, upon careful inquiry, wrong of the uterus. There is nearly always more or less leucorrhœa, and the discharge is frequently of an offensive, irritating character. The internal administration of eupatorium, alternated or combined with hydrastis, will always help in such cases, and they will many times accomplish everything desired.

℞ Mother tinct. eupatorium aromaticum............ ʒ ij,

Fluid hydrastis............................ ʒ ij,

Water.................................... ℥ iijss.

M. S. One teaspoonful every hour.

"The frequent repetition of the dose in a fluid form renders other local uses of these drugs unnecessary. Other remedies may be required oceasionally, but it is even surprising to see how rapidly some cases of nursing sore mouth heal under the influence of this simple prescription. The burning mouth and tongue are cooled, the leucorrhœal discharge is modified, lessened, and, not infrequently, entirely stopped; and the nervous element of the disease, characterized by morbid watchfulness, throbbing headache, etc., is perfectly controlled in most cases. Eupatorium is said to be a remedy for nervousness, but we have never observed that its virtues were very marked in this regard except in this terrible disease, so frequently met with in nursing women, but here it certainly is a first-class remedy."

**Boils.**—Dr. John Aulde, following the suggestions of Dr. Sidney Ringer, has met with most satisfactory results by adopting the following plan. The diet is to be regulated, and if constipation exists a teaspoonful of magnesia sulph. in a glass of cold water should be taken every morning before breakfast:

℞ Calcii sulphidi............................grs. iij,
Sacch. lactis..............................grs. xxx,
Misce bene et div. in chart., No. xxx.

Sig. Five powders daily at intervals, between meals.

By this method beginning boils will be aborted, and those far enough advanced to threaten a siege of several weeks and successive crops will soften and heal in such short time that the patient will be surprised at the result. When they can be obtained, granules containing one-tenth grain are to be preferred to the powders. The urine should be examined for sugar, as boils and diabetes often go together.—*Summary.*

**Liquor Hydrargyri Bichloridi in Dysentery.**—Dr. March, in the London Medical Times and Gazette, declares that half-minim doses of the liquor hydrargyri bichloridi (B. P.), administered hourly, speedily and infallibly relieves all forms of dysentery except infantile and tropical. Dr. Ringer, several years ago, recommended bichloride of mercury in doses of 0.01 grain, repeated hourly, and the writer has obtained most excellent results from the remedy.—*South. Clinic.*

**Sorghum and Typho-Malarial Fever.**—A writer in Nashville Med. Journal thinks that Typho-Malarial Fever, so-called, is produced by eating the syrup of the Chinese sugar cane. We do not agree with him, but give the following extract from his paper: He says, "We have many facts that we might educe, which would show conclusively that it cannot be typhoid, typhus, nor pernicious fever, as it is called by some.

"Now, in looking for a cause, I wish to invite the attention of the profession to some observations I have made in regard to this fever in my community. I first noted the fact that the families in which the fever prevails are those that use the product of a plant brought from China a few years ago, sorgo sucre, or Chinese sugar cane, and the families that have never used it have escaped entirely. Now, I have not known a well-authenticated case, except in families that have used sorghum pretty generally for, at least, a year or more preceding the attack.

"So, when we inquire for the history of the plant, we find that it was first introduced into this country, by a gentleman who was laboring in the interest of the U. S. Patent Office in the year 1854, from France, into which country it had been introduced, probably three years before, from the north of China.

"So we see the introduction of this plant into our country, and the first authentic account of this fever prevailing in the United States are coeval or nearly so. Now, I would like to know if the above facts are worthy of the attention of the profession in the investigation of the subject, and if they can be corroborated by the opinions of others."

**Paraldehyde as a Hypnotic.**—Paraldehyde, the newer addition to our list of hypnotics, seems to be growing in favor in Germany. A very extended series of experiments seems to justify the claim that it is among the remedies best adapted to the production of sleep in cases in which there is no inflammatory or organic disease of the brain or in which wakefulness is not the direct result of pain. The drug is not an anodyne or a narcotic (using these terms in their accepted sense), but a hypnotic, pure and simple. It seems to produce a more natural sleep than follows the exhibition of chloral hydrate, the sleep being quiet, refreshing, lasting from three to eight hours, and being followed by none of that fullness or pain in the head which are the bane of narcotics as a class. The remedy seems to be particularly adapted to cases of simple insomnia, due to nothing more grave than mental worry. The dose, as recommended, ranges from half a drachm to a drachm and a half. No untoward symptoms have been reported from the larger quantity. The following formula is recommended as effectual in overcoming the disagreeable after-taste of the drug:

℞ Paraldehyde.................................*m* xxx,

Aquæ.......................................℥ jss,

Syr. aurantii corticis.........................ʒ ij,

Spiritus chloroformi...........................*m* xxx.

M. —*Med. Age.*

**Injections of Glycerine and Carbolic Acid for the Preservation of Bodies for Dissection.**—Dr. O. T. Freer, of Munich, in the Chicago Medical Journal and Examiner, says: "During a course of dissection in the anatomical department of the University of Munich, I observed that the material showed a remarkable resistance to decay, being still fresh at a period when the average subject of Chicago's dissecting rooms is too much decomposed for use. The parts which I was dissecting were in almost as perfect a condition after exposure to the air of a warm room for from two to four weeks, as when they were first given me: and those portions from which the skin had not been removed remained entirely unchanged.

On inquiry, I learned from Prof. Rudinger, Munich's anatomist, a man who has written numerous valuable works and done much for science by his original researches, that he preserved his subjects by injecting into the femoral artery a mixture of glycerine, carbolic acid and alcohol, a method invented by himself and used here since 1872.

Since its introduction he had seen no bad effects from dissection wounds here.

He said that, according to the amount of injection-material used, subjects would keep from two to six months in a summer-temperature, the only necessity being moisture to prevent mummification.

The solutions used by Prof. Rudinger are:

(*a*) For preserving bodies for long periods, three to six months: Glycerine, forty parts; carbolic acid, crystalized, eleven; alcohol, eight.

(*b*) For preserving bodies from two to three months: Glycerine, eighty parts; carbolic acid, seventeen; alcohol, thirteen.

The amount injected varies according to the length of time it is desired to preserve the subject, from two to four liters being ordinarily used; but an average body will readily contain six and more.

The advantages of this method are that the tissues preserve their natural appearance and pliancy from the commencement of an ordinary dissection to the end. Students, therefore, need not hurry their dissection, even in warm weather, as decomposition is so slow that the parts dissected last are in as perfect a state as those seen first. The solutions mentioned above do not whiten the tissues, which retain their natural appearance to the end."—*Maryland Med. Jour.*

**Warts.**—Dr. Cordes, of Geneva, states that he has always found the following application successful: Iodine six, crystallised carbolic acid twenty-one, and alcohol two parts and a half by weight. After scraping the wart or cutting it down to a level with the skin (without causing it to bleed), he touches the wart with a few drops of the above solution. In a minute it becomes soft, and allows of another scraping and a new application; and sometimes even a third scraping and application can be made without causing bleeding.—*Medical Times, Nov. 24, p. 609.—Braithwaite's Retrospect.*

**Escharotics.**—Esmarch's painless caustic powder, for the removal of warts, tumors, etc., is composed of:

| | | |
|---|---|---|
| ℞ | Arsenious acid | part 1 |
| | Sulphate of morphia | " 1 |
| | Calomel | " 8 |
| | Pulv. gum arabic | " 48 |

This is to be sprinkled on the cuticle daily. The surface should be denuded either with the knife or a blister. Canquoin's paste, for the same purpose, is made, according to M. Charles, by the following formula:

| | | |
|---|---|---|
| ℞ | Chloride of zinc, fused | parts 10 |
| | Alcohol, 60° | " 2 |
| | Wheat flour | " 15 |

Rub the zinc chloride to a fine powder, add the alcohol, rub again and incorporate the flour, strongly pressing with the pestle. As soon as the paste is homogeneous, spread with a roller or bottle into sheets about one-eighth of an inch thick, and after a few hours put into a well-corked bottle.

Latour's nitro-chloride of zinc paste, a most excellent escharotic, is made by dissolving fifty parts of chloride of zinc and one hundred parts of nitrate of zinc in eighty parts of water. The solution is made by the aid of heat. When it cools, add to each one hundred parts of the fluid seventy-five parts of wheat flour, and incorporate as in Canquoin's paste.—*St. Louis Druggist.*

**Nævus.—Local Application of Liquor Arsenicalis.**—The ordinary treatment of nævus appears so severe that mothers naturally object to it (the knife, for example, or even puncture or vaccination). I was, therefore, led to try what arsenic would do as a local remedy, and in my hands it has succeeded admirably, my last eight cases having been cured perfectly and painlessly by the local application of this remedy. The preparation I use is the ordinary liquor arsenicalis of the Pharmacopœia, with which the nævus is to be painted night and morning until ulceration takes place; and I find that the cure is effected in from three to five weeks.—*Mr. W. J. Beatty, Stockton-on-Tees, British Medical Jour., Nov. 24, p. 1016.—Braithwaite's Retrospect.*

**Benzoate of Soda in Infantile Diarrhœa.**—Dr. R. Guaita (Revista Italiana de Terap. e Igiene, Gennaio, 1884) thinks the summer diarrhœa of children is, for the most part, a zymotic disease, and due, principally, to a special ferment (microbe), which either comes from without, or is developed during the process of digestion from certain kinds of food. It occurred to him, therefore, that benzoate of soda, recommended by Kapuscinsky and Gilewicz, would prove valuable in these cases, being anti-fermentative. It may be administered alone or in combination with bismuth.

This treatment was tried in fifty-three cases, the children being from six months to two years old. In thirty-five cases the affection had lasted from twenty four to thirty hours; in the remaining eighteen, from six to fourteen days. In the thirty-five cases the cure was complete in from four to eight days; in the eighteen, the average time was twenty-one days. The treatment is preceded by an efficacious purge—calomel or jalap: this having acted, the soda treatment is commenced.—*Gazz. degli Ospitali.*

**Vanillism.**—Drs. Layet and Arnozan have communicated to the French Association for the Advancement of Science the results of their researches on the physical qualities, the effects and the parasites of vanilla. They cited several cases of poisoning by eating of vanilla ice-cream. It was at first supposed to be due to the formation of lactate of tin in the vessels, but cases of poisoning followed the use of the same stock of vanilla in the hands of a confectioner in another city, who had purchased it. There were other cases in Berlin.

The symptoms simulated cholera: continued vomiting, diarrhœa, epigastric pain, cramps in legs, face and extremities cold and purple. The symptoms ensued in about two hours after eating, and recovery in three or four hours.

Among operatives, those who cut the vanilla into small pieces are troubled with papular eruptions of the face and hands, with local heat and swelling, irritation of the eye and lids, and oftentimes patches of redness and desquamation.—*Boston Med. and Surg. Jour.*

**Disinfectants.**—Dr. W. E. Buck writes to the British Medical Journal: Most practitioners must have often realized the inefficiency of disinfectants in allaying the fœtor of cancerous ulcers. an annoyance which sometimes troubles the patient even more than the pain or the thought of death. After failure with the whole round of disinfectants, I tried a saturated solution of hyposulphite of soda added to an equal quantity of water and found it exceedingly efficacious. The ulcerating surface was well syringed and washed with the solution, and was then covered with rags steeped in the solution. Most disinfectants seem to lose their virtue after a few days' application, but I have used this for months in the same patient with continuous good effects. It is cleanly, has no smell, does not stain, and is very cheap.—*South. Clinic.*

**Consumption from Street Dust**—Mon. S. Vignal has concluded that the germ of consumption may lurk in the dust of rooms or streets, as he has proven that the bacilli in the matter expectorated by consumptive patients are not destroyed by being repeatedly dried and moistened, and dust containing such matter may be an agent of contagion to persons predisposed to the disease, or in whom the bacilli find suitable soil for propagation. A guinea pig inoculated with the matter in the condition in which it is likely to exist in the streets died in three months from genuine consumption.—*The Drugman.*

